# An Overview of the Spices Used for the Prevention and Potential Treatment of Gastric Cancer

**DOI:** 10.3390/cancers16081611

**Published:** 2024-04-22

**Authors:** Katarzyna Kostelecka, Łukasz Bryliński, Olga Komar, Justyna Michalczyk, Agata Miłosz, Jan Biłogras, Filip Woliński, Alicja Forma, Jacek Baj

**Affiliations:** 1 Department of Anatomy, Medical University of Lublin, ul. Jaczewskiego 4, 20-090 Lublin, Poland; katarzyna.k@vp.pl (K.K.); lukbry2@gmail.com (Ł.B.); olgakomar720@gmail.com (O.K.); justynaelwiramichalczyk@gmail.com (J.M.); agatamilosz95@wp.pl (A.M.); janbilogras@gmail.com (J.B.); jacek.baj@umlub.pl (J.B.); 2 Department of Forensic Medicine, Medical University of Lublin, ul. Jaczewskiego 8b, 20-090 Lublin, Poland; rush22235@gmail.com

**Keywords:** gastric cancer, ginger, garlic, turmeric, black cumin, chili pepper

## Abstract

**Simple Summary:**

Despite the significant improvements in the diagnosis and therapeutic strategies for gastric cancer, this malignancy still remains one of the most prevalent worldwide, with a significant mortality rate. Recently, the number of studies concerning herbal medicine and its use in various cancers has increased significantly. For example, there has been research focusing on its applications alone or in combination with other therapeutic strategies, such as chemotherapy. Therefore, because of the continuous research on newer spices, we aimed to summarize the current knowledge regarding the application of herbal medicine in gastric cancer patients, taking into account their potential as a part of potential cancer therapy. Besides providing a summary of the potential alternative therapeutic approaches for gastric cancer, the findings of this paper might provide insight into further research directions.

**Abstract:**

Gastric cancer (GC) ranks third in terms of cancer-related deaths and is the fifth most commonly diagnosed type of cancer. Its risk factors include *Helicobacter pylori* infection, *Epstein–Barr virus* infection, the consumption of broiled and charbroiled animal meats, salt-preserved and smoke-enhanced foods, alcohol drinking, tobacco smoking, exposure to ionizing radiation, and positive family history. The limited effectiveness of conventional therapies and the widespread risk factors of GC encourage the search for new methods of treatment and prevention. In the quest for cheap and commonly available medications, numerous studies focus on herbal medicine, traditional brews, and spices. In this review, we outline the potential use of spices, including turmeric, ginger, garlic, black cumin, chili pepper, saffron, black pepper, rosemary, galangal, coriander, wasabi, cinnamon, oregano, cardamom, fenugreek, caraway, clove, dill, thyme, *Piper sarmentosum*, basil, as well as the compounds they contain, in the prevention and treatment of GC. We present the potential molecular mechanisms responsible for the effectivity of a given seasoning substance and their impact on GC cells. We discuss their potential effects on proliferation, apoptosis, and migration. For most of the spices discussed, we also outline the unavailability and side effects of their use.

## 1. Introduction

**Figure 1:**
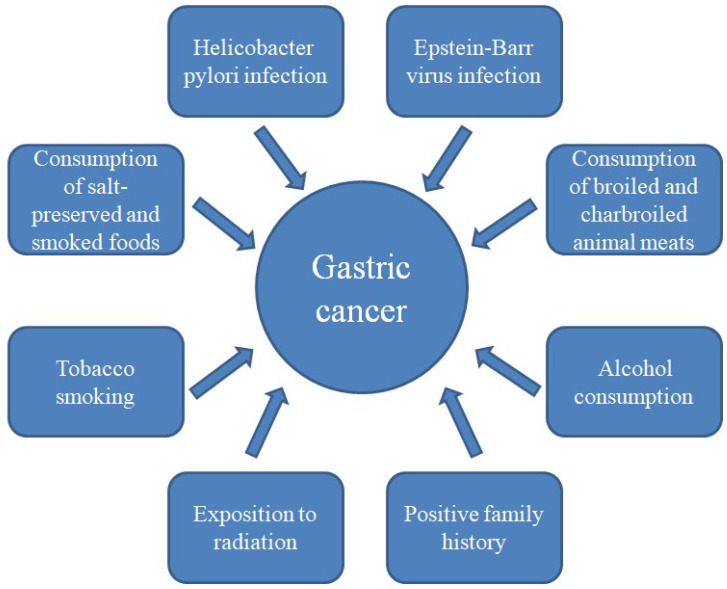
The potential factors that increase the risk of GC.

Gastric cancer (GC) causes 1 in every 12 deaths globally. According to Global Cancer Statistics for 2018, GC is responsible for over 1,000,000 new cases and 783,000 deaths worldwide. It ranks third in terms of cancer-related deaths and is the fifth most prevalent type of cancer to be diagnosed. GC usually appears after age 60. Men are two to three times more susceptible than women. Incidence rates in Northern America, Northern Europe, and African regions are generally low; GC is noticeably more frequent in Eastern Asia [1,2]. The etiology of GC is multifactorial (Figure 1) [3].

It has been estimated that more than 50% of the population worldwide carries the *Helicobacter pylori* (*H. pylori*) infection. The bacterium is capable of causing precancerous multifocal anthropic gastritis [4,5]. It can trigger the generation of reactive oxygen species (ROS), which can damage DNA and cause mutations and the hypermethylation of promoter CpG islands, which affects genes that prevent carcinogenesis, like the RPRM and TWIS1 [6]. Furthermore, virulent strains of *H. pylori* can carry the cytotoxic-associated gene A (CagA). In vivo, a protein produced based on the CagA gene disrupts intercellular junctions, decreases the polarity of epithelial cells, reduces apoptosis, and increases the proliferation of gastric cells. There are many more factors produced by *H. pylori* that may play a pivotal role in carcinogenesis (e.g., vacA s1/m1, vacA s1/m1/i1, or BabA) of GC [7,8].

Almost 10% of all GC can be related to Epstein–Barr virus (EBV) infection. In classification introduced by The Cancer Genome Atlas in 2014, this type of tumor was named EBV-associated gastric cancer (EBVaGC). EBVaGC is usually found in cardia and has a moderate or poor degree of differentiation and a better prognosis than EBV-negative GC [9].

The consumption of broiled and charbroiled animal meats and salt-preserved and smoked foods enhances GC progression. At the same time, fresh fruit and vegetables are considered protective. Also, the intake of N-nitroso compounds found in tobacco smoke, chewing tobacco, and beer have been linked to GC. Moreover, potent amplifiers of GC carcinogenesis are ethylene alcohol and tobacco smoking [10]. A higher risk of GC applies to some occupations, such as farmers, carpenters, tin miners, and steel workers. Exposure to ionizing radiation may also contribute [11].

Although only 10% of GCs show family aggregation, a positive family history is considered one of the most important risk factors. Hereditary diffuse GC resulting from cadherin 1 (CDH1) gene alternations is the most well-known type of familial GC. What is more, several studies documented an association between polymorphisms of genes involved in inflammatory response and GC carcinogenesis [4,10].

The standard treatment for localized GC is surgical excision (endoscopic in early GC or subtotal/total gastrectomy with D1/D2 lymphadenectomy) with perioperative and postoperative chemotherapy. Novel preoperative therapies (still studied) include the addition of anti-human epidermal growth factor receptor 2 (anti-HER2) and anti-vascular Endothelial Growth Factor (anti-VEGF) drugs, such as trastuzumab and pertuzumab, to standard FLOT protocol or use of the Programmed death-ligand 1 (PD-L1) inhibitors [12,13], and these are recommended for patients who undergo upfront surgery and T3-T4 grade adjuvant chemotherapy. Adjuvant chemotherapy is recommended for patients who undergo primary surgery with stage II/III disease. In the case of unresectable/metastatic GC, the first line of treatment is chemotherapy, sometimes in combination with immunotherapy [14,15].

The limited effectiveness of conventional therapies and the widespread risk factors of GC encourage the search for new methods of treatment and prevention. In the quest for cheap and commonly available medications, numerous studies focus on herbal medicine, traditional brews, and spices.

## 2. Spices for the Prevention and Potential Treatment of GC

Spices with potential use in the prevention of GC are turmeric, ginger, garlic, black cumin, black pepper, galangal, coriander, wasabi, oregano, fenugreek, caraway, clove, dill, thyme, and *Piper sarmentosum* (Table 1). Spices with potential for treatment include turmeric, ginger, garlic, black cumin, chili pepper, saffron, black pepper, rosemary, galangal, coriander, wasabi, cinnamon, oregano, fenugreek, caraway, clove, dill, thyme, and basil (Table 2).

### 2.1. Tumeric

Tumeric is one of the species that had potential anticancer activity; turmeric includes curcumin. According to Zhend et al. [43], this polyphenolic compound could be applied in the treatment of nasopharyngeal cancer, lung cancer, hepatobiliary cancer, breast cancer, colorectal cancer, prostate cancer, cancer of the uterus, hematopoietic tumors and GC [43]. Due to its hepatoprotective, hepatic steatosis-inhibiting, anti-fibrotic effects, curcumin is also a potential therapeutic option in the prevention and treatment of liver and biliary tract diseases [123].

The potential anticancer activity of curcumin is associated with inhibiting proliferation and migration, inducting the apoptosis of GC cells, which can be reached by different pathways [124]. One of them is the inhibition of the expression of sonic hedgehog homolog (Shh), GLI Family Zinc Finger 1 (Gli1), and Forkhead box protein M1 (Foxm1) in the Shh signaling pathway and the inhibition of the expression of β-catenin in the Wnt signaling pathway. In this mechanism, curcumin suppresses the Shh and Wnt signaling pathways, which is associated with lower ability proliferation, migration, and invasion of cancer cells [57]. At the same time, the research by Hongbing et al. carried out on cell lines showed that curcumin may activate the P53 signaling pathway and inhibit the tophosphatidylinositol-3 kinase (PI3K) signaling pathway [58]. Curcumin also can affect the epigenetics of GC. In the study from 2019, curcumin presented a histone-modulating mechanism based on the regulation of histone acetylation and deacetylation enzyme activity. By the inhibition of histone deacetylases (HDACs) and *histone* acetyltransferases (*HATs*), curcumin suppresses proliferation and induces the apoptosis of GC cells [59]. The other epigenetic modification is the expression of MicroRNA34 (miR34). The downregulation of mir34 expression may be engaged in GC development: a lower level of miR34 in GC cells promotes proliferation and inhibits apoptosis [60]. Curcumin could significantly increase the level of miR34. The study carried out on cell lines showed that the elevation of the miR34 level inhibits proliferation and induces the apoptosis of GC cells [61]. MiR33b is also involved in GC tumorigenesis; in this case, curcumin, by increasing the expression of this molecule, stimulates GC cell apoptosis [62]. On the other hand, miR21 is a molecule, and its increased levels can be engaged in GC development. A study from 2018 showed that curcumin decreases its expression and has an antitumor effect [63].

Curcumin also seems to be usable in the prevention of GC. Its anti-inflammatory, antioxidant, and protective effects may be useful in inhibiting damage to the gastric mucosa by various agents such as non-steroidal anti-inflammatory drugs (NSAIDs) and stress bleeding [16,17]. Moreover, curcumin has antibacterial properties, which are associated with the inhibition of HP infection [18,19]. The study carried out on the mice model showed that curcumin downregulates the expression of inflammatory cytokines and chemokines as toll-like receptors (TLRs) and (myeloid differentiation primary response 88) MyD88 [125].

One of the biggest advantages of curcumin is its safety. The studies carried out on humans and animals showed that turmeric and curcumin are nontoxic [126,127]. Despite this, its bioavailability is a problem. The problem with its poor solubility, low absorption, rapid metabolism, and rapid systemic elimination poses a threat to its therapeutic potential [128]. Therefore, improving bioavailability is important. One of the proposed methods is N-carboxymethyl chitosan (NCC)-coated curcumin-loaded modified solid lipid nanoparticles (NCC-SLN), by which, through the mechanism of inhibition, the rapid release of curcumin in an acidic environment enhances the bioavailability of curcumin [129]. The other option is the curcumin in casein nanoparticles (CasNPs), which also improves the bioavailability of curcumin [130]. Ligand-modified curcumin liposomes are a promising method for increasing the bioavailability of curcumin. Their use not only increases the bioavailability of curcumin but also improves the therapeutic effect [131]. Future research should focus on improving the bioavailability of curcumin [132].

### 2.2. Ginger

Ginger is a herbaceous plant known since ancient China. As many as 400 bioactive compounds have been isolated, some of which have anticancer properties [133,134].

One of them is a phenolic compound, 6-gingerol, which is the main active compound isolated from ginger. In the study by Youjun et al. [64], 6-gingerol showed potential usefulness in increasing the radiosensitivity of GC cells. The effect of 6-gingerol was to inhibit GC cell proliferation, enhance IR-induced cell arrest at the G2/M stage, and induce apoptosis. Moreover, due to its potential ease of obtaining and widespread occurrence, it seems that 6-gingerol may have potential use as a radiosensitizer, but further research is needed [64]. The effect of 6-gingerol on cisplatin chemotherapy was also investigated. It was shown that 6-gingerol increased the sensitivity of GC cells to cisplatin by arresting the cells at the G1 stage. Moreover, the use of 6-gingerol inhibited the migration ability of GC cells, and the combination of cisplatin and 6-gingerol caused not only the inhibition of migration but also the proliferation of cancer cells [65]. Furthermore, in a study carried out on rats, ginger extract and ginger juice appear to be useful in alleviating nausea and vomiting and the inhibition of gastric emptying induced by cisplatin treatment [66]. The other compound, which can be found in ginger and has a potential anticancer property, is 8-paradol. This compound induces the apoptosis of cancer cells by promoting the mitophagy of cells with promoted PINK1/Parkin-associated mitophagy. 8-paradol also inhibits GC cell proliferation [67].

Ginger is also used in the prevention of GC. Steamed ginger extract showed anti-inflammatory effects: it inhibited the synthesis of interleukin (IL)-8, tumor necrosis factor α (TNF-α), IL-6, inducible NOS (iNOS), and interferon-gamma (IFN-γ); inhibited the Nuclear factor kappa B (NF-κB) signaling pathway; and also reduced the production of nitric oxide and activity myeloperoxidase in gastric epithelial cells infected with *H. pylori*. Due to this, ginger extract can inhibit acute gastritis, chronic inflammation of the mucosa and submucosa, cryptitis, as well as the degeneration and erosion of epithelial cells caused by *H. pylori* [20,21]. Ginger extract has a synergistic effect with clarithromycin and has also shown activity against antibiotic-resistant strains of *H. pylori* [22].

Information on the bioavailability of compounds contained in ginger is limited. After administration, they are quickly absorbed, accumulate in many tissues, and are intensively metabolized, which is why they are excreted in the form of metabolites in bile and urine [135]. An increase in the absorption of compounds contained in ginger extract can be achieved using the solid dispersion method [136]. Another option is to use castor oil as a nanostructured lipid carrier, which improves the bioavailability of ginger extract [137]. Ginger is described as safe to use and does not cause side effects [135].

### 2.3. Garlic

The health-promoting properties of garlic have long been known. The beneficial properties of garlic may be related to its immunomodulatory and anti-inflammatory effects, which are achieved through the stimulation of macrophages, lymphocytes, NK cells, dendritic cells, and eosinophils, as well as through mechanisms including the modulation of cytokine secretion, immunoglobulin production, phagocytosis, and macrophage activation. Garlic also exhibits antimicrobial, anti-arthritic, anticoagulant, anticancer, hypoglycemic, and hypolipidaemic effects.

The compounds it contains also have potential applications in the prevention and treatment of GC [68,69]. One of them is allicin. Its mechanism of action is to induce the apoptosis of GC cells in the mechanism of release of cytochrome from mitochondria, the hydroxylation of caspases, and the activation of the p38 mitogen-activated protein kinase (MAPK)/caspase 3 pathway [70,71]. The action of diallyl disulfide is associated with the arrest of GC cells in the G2/M phase of the cell cycle, induction of apoptosis, and inhibition of proliferation [72,73]. Another compound, diallyl trisulfide, also exhibits antitumor properties: it suppresses tumor growth and induces cell apoptosis in the mechanism of the activation of AMP-activated protein kinase (AMPK) [74]. Another compound is s-allilocysteine. It may find use in gastric ulcer healing due to its ability to inhibit the activation of inflammatory mediators: cyclooxygenase-2, prostaglandin E2, IL-1β, TNF- α, IL-6 [75,76].

Garlic supplementation appears to have a beneficial effect on reducing the risk of death from GC [77]. Interestingly, a 2020 study carried out on 3365 residents of a high-risk region for GC found that garlic supplementation had a beneficial effect on GC mortality, but only among non-drinkers [78]. At the same time, a study from 2012 showed that garlic supplements were associated with non-statistically significant reductions in GC mortality [79].

There is a lack of strong evidence regarding the use of garlic in the prevention of GC. On the one hand, according to a meta-analysis by Kodali et al. [23], the consumption of garlic has been shown to have a beneficial effect in the prevention of GC, but this is dose-dependent: higher garlic consumption provides a greater degree of protection. Moreover, easy availability and good taste make garlic consumption an easy method of GC prevention [23]. At the same time, the study by Hanseul et al. [24] found that garlic consumption reduces the risk of GC and also has no effect on *H. pylori* infection. Further research is needed to investigate the impact of garlic consumption in preventing GC [24].

Garlic does not present any side effects, but its drawback may be its intense smell. However, this can be overcome by using garlic-based supplements. Other advantages are its easy availability and good taste [23].

### 2.4. Black Cumin

Black cumin is well-known for its culinary uses, as well as its antioxidant, anti-inflammatory, immunomodulatory, anticancer, neuroprotective, antimicrobial, antihypertensive, cardioprotective, antidiabetic, gastroprotective, nephroprotective, and hepatoprotective properties, showing its potential medicinal value [138].

Black cumin contains thymoquinone, which has shown anticancer activity against GC. This compound can inhibit proliferation and induce apoptosis of GC cells. A 2017 study conducted on GC cell lines showed that thymoquinone inhibits the expression of key proteins of phosphatidylinositol-4,5-bisphosphate 3 kinase/protein kinase B/ mechanistic target of rapamycin (PI3K/Akt/mTOR) pathway [80]. Similar results were obtained in a study from 2023 carried out on the mouse model [81]. Another mechanism by which thymoquinone affects GC cells is the signal transducer and activator of the transcription 3 (STAT3) pathway. The inhibition of constitutive STAT3 phosphorylation resulted in the inhibition of the protein expression of STAT3 target gene products, such as survivin, cyclin-D, VEGF, and Bcl-2 (B-cell lymphoma 2), and the increased expression levels of Bax (Bcl2-associated X protein); this induced the apoptosis of GC cells [82]. Thymoquinone may also potentiate the effects of 5-fluorouracil (5-FU). In a study by Xiaofei et al. [83], it was shown that thymoquinone sensitizes GC cells to 5-FU, which enhances their apoptosis [83].

Black cumin also shows activity against *H. pylori* infection. A 2016 study used black cumin seeds in the eradication of *H. pylori* infection. Results showed that ground seeds at a dose of 2 g/d given together with 40 mg/d of omeprazole showed clinical activity against *H. pylori*, which may be comparable to the activity of triple therapy [25]. The effect of black cumin (6 g/day) in combination with honey (12 g/day) was also investigated. This combination also showed activity against *H. pylori* [26].

Despite its potential role as a compound found in black cumin, thymoquinone’s drawback is its poor water solubility, bioavailability, and stability [139]. The use of black cumin is associated with low toxicity, and the use of black cumin in the recommended doses is safe. Oral use has no adverse effect on liver or kidney function. The use of high doses over a long period and topically may lead to dermatitis; the use of high doses of 50–100 mg/kg drastically reduces the glutathione (GSH) concentration [140].

### 2.5. Chili Pepper

The chili pepper is a common spice used to enhance the flavor of food. It owes its characteristic pungent taste to the presence of capsaicin [141]. The effect of both this compound and chili peppers on GC is inconclusive. A 2014 study on GC cells showed that capsaicin inhibits proliferation and induces apoptosis of GC cells in a mechanism that modulates the expression of apoptosis-regulating proteins: it acts on the production of caspase-3 and reduces the expression of Bcl-2. In addition, it reduces the expression of phosphorylated extracellular signal-regulated kinase 1/2 (ERK 1/2), p38 MAPK, or c-Jun N-terminal kinase (JNK) [84]. An antitumor effect can also be obtained by affecting histone acetylation. This epigenetic effect is achieved by restoring the activity of hMOF HATs, resulting in the inhibition of GC cell proliferation [85].

On the other hand, the consumption of chili peppers may be a potential risk for GC; a meta-analysis by Lei et al. [86] showed a positive correlation between the consumption of significant amounts of chili peppers [86]. Similar results were obtained in the study from 2003: the consumption of large amounts of capsaicin (90–250 mg of capsaicin per day) may be an independent factor in GC [87]. In a case–control study, it was also shown that people who consumed chili peppers had a higher risk of GC than non-consumers [88]. The opposite conclusion was reached by Changchang et al. [142]; in their meta-analysis, they found no association between chili pepper consumption and increased GC risk [142]. The effect of capsaicin on the development of GC requires further research. They need to take into account other carcinogens in the diet and environment and the use of capsaicin of known purity [143].

The potential use of capsaicin is also hampered by its poor bioavailability (capsaicin has a short biological half-life in plasma and is rapidly eliminated from the body) and poor water solubility. In addition, it can cause several side effects: skin redness, painful hypersensitivity, nausea, intense tearing of the eyes, conjunctivitis, eyelid spasm (prolonged, forced, involuntary closing of the eyelids), vomiting, abdominal pain, stomach cramps, bronchospasm, and burning diarrhea in patients [144].

### 2.6. Saffron

Saffron is extracted from the dried flowers of the crocus (*Crocus sativus* L.) and used in the kitchen as a spice that imparts color, flavor, and aroma to food and drinks but is also credited with medicinal properties [145].

The main constituents contained in saffron are crocins (glycosidic derivatives of crocetin), picrocrocin (responsible for the bitter taste), and safranal, which is formed by the dehydration of picrocrocin during storage, giving the spice its characteristic aroma [146,147,148].

Given the reports of saffron’s medicinal properties, whether acting as an antioxidant or inducing an inhibitory effect on cancer cells [149], it has also been studied for its anticancer effects in GC. In a study conducted on rats with previously induced GC treated with saffron aqueous extract (SAE) by intraperitoneal (IP) injection for 50 days, it was proven that plasma antioxidant activity increased after SAE administration in a dose-dependent manner. SAE treatment also reduced serum lactic acid dehydrogenase (LDH) levels and, as a result of higher doses of SAE, induced apoptosis in GC tissue. Furthermore, a pathomorphological study showed that SAE treatment significantly reduced the histological severity of Methyl-N-Nitro-N-Nitrosoguanidine (MNNG)-induced lesions in the gastric mucosa [150]. Other studies have already focused on specific saffron constituents and their antitumor effects. One of these is crocin, which has shown an inhibitory effect on GC cell proliferation. The inhibition of GC cell proliferation increased with a higher dose of crocin. Crocin was also found to reduce the expression of TPM4, whose increased expression is found in GC. However, the overexpression of TPM4 abrogates the inhibitory effect of crocin on tumor cell proliferation. Therefore, with concomitant treatment with crocin, it is worthwhile to use Knockdown TPM4, which will enhance the inhibitory effect on tumor cell proliferation [89]. Another study also confirmed the inhibitory effect of crocin on GC cell growth; crocin also increased Bax expression and decreased Bcl-2 expression in GC cells. The increase in the Bax/Bcl-2 ratio after crocin treatment indicates the stimulation of apoptosis [90]. A study in rats also confirmed the ability to promote apoptosis as well as inhibit proliferation in MNNG-induced GES-1 cells. Crocin was also shown to protect via the Nrf2/Hippo signaling pathway against MNNG-induced malignant transformation [151]. Another study, using human GC cell lines, showed a crocin-induced decrease in Krueppel-like factor 5 (KLF5) and hypoxia-inducible factor 1-alpha (HIF-1α) expression, which is increased in GC tissues and cells. Interestingly, crocin decreases KLF5 expression by increasing miR-320 levels. Unfortunately, increasing KLF5 expression impairs crocin function and increases HIF-1α expression. In contrast, crocin treatment also led to a significant reduction in the number of migrating and invasive GC cells [152].

In addition to crocin, crocetin was also analyzed. A study in 30 rats showed a significant dose- and time-dependent inhibition of GC cell proliferation following crocetin administration. After treatment with crocetin, serum antioxidant capacity increased, and LDH activity decreased. A histopathological study showed that tumor lesions in the stomach tissue of crocetin-treated rats were significantly reduced [91]. Another study showed that crocetin may have an inhibitory effect on angiogenesis. The study also observed that crocetin inhibited cell proliferation and migration, affecting vessel formation [92].

The side effects of saffron have not yet been described in GC, but when used, for example, in a study for the treatment of depression, anxiety, and other psychiatric disorders, the following were mentioned: nausea, decreased appetite, anxiety, and headache. With the use of crocin, manometric hemorrhage, dyspnea, and agitation were present [153].

To assess the toxicity of saffron, the toxicity of the saffron component safranal was investigated in mice and rats. Weakness, anorexia, decreased food and water intake, and weight loss were observed, which were significant at higher doses. On microscopic examination of the organs, abnormalities appeared in the kidneys, where edema and cytolysis were found. Progressive emphysema and lymphocyte infiltration were found in the lungs. Hematological studies showed a significant decrease in red blood cells, hemoglobin, hematocrit, and platelets in the treatment groups. However, in biochemical parameters, there was an increase in LDH and serum urea nitrogen (BUN) levels in the treatment groups and a decrease in total cholesterol, triglycerides, and alkaline phosphatase (ALP). Safranal showed greater toxicity after intraperitoneal administration compared to oral administration, which may be due to the greater first-pass effect and lower absorption rate during oral treatment [154]. In contrast, a 2013 study showed serious side effects, a decrease in amylase activity, a shortening of PTT, and a decrease in MXD levels (monocytes, basophils, and eosinophils) [155]. In another safety assessment of saffron tablets in healthy volunteers, apart from changes in some hematological parameters (reduction in red blood cells, hemoglobin, hematocrit, and platelets) and biochemical parameters (increase in sodium, blood urea nitrogen, and creatinine) further within normal limits, no serious side effects were shown [156].

### 2.7. Black Pepper

Black pepper, known as the ‘king of spices’, is obtained by drying the unripe fruit of *Piper nigrum L*. Both whole and lightly crushed peppercorns are used in cooking to improve the flavor of dishes [157].

The main active ingredient in pepper is alkaloid piperine (PIP), which is responsible for the pungent taste and also exhibits several medicinal properties such as antioxidant, anticancer, and anti-inflammatory effects [158]. PIP has shown potential for use in colorectal cancer [159], breast cancer, and melanoma [160]. Our review will focus on its use in GC.

The effect of PIP on cell proliferation and apoptosis has been investigated. It was proven that a higher dose of PIP caused an increase in the rate of inhibition of cell proliferation and also increased apoptosis. PIP treatment promotes the production of intracellular reactive oxygen species. Their excess can induce the apoptosis of cancer cells. Furthermore, PIP decreases the mitochondrial membrane potential, indicating a link between its pro-apoptotic effect and mitochondrial apoptosis. After treatment, a decrease in the expression of Bcl-2, a key protein of the mitochondrial apoptosis pathway, was also observed, with a concomitant increase in the expression of Bax. PIP also showed an effect on other important proteins of the mitochondrial apoptosis pathway, caspase-3 and caspase-9, by increasing their levels [161]. Another study confirmed the inhibition of proliferation and the induction of apoptosis of human GC cells by PIP through the inhibition of the PI3K/Akt signaling pathway. Piperine increased caspase-3 activity in GC cells. In a study of mice treated with PIP treatment, the inhibition of heterotopic tumor growth was noted. Additionally, PIP showed no toxicity during the 18-day treatment [93].

PIP also showed anti-inflammatory effects. In a study, piperine was found to inhibit IL-6-induced IL-1β expression via the inhibition of STAT3 and p38 MAPK activation. GC is characterized by a high expression of IL-6, and this high IL-6 production positively affects the aggressiveness of GC and also reduces the prognosis of patients. Because IL-6 increases cell invasiveness, piperine, by downregulating IL-6 expression, will counteract this invasion [94]. PIP has also shown anti-inflammatory effects in chronic gastritis in Mongolian gerbils, which was caused by *H. pylori*, one of the causative agents of GC. This study demonstrated that PIP reduces the number of *H. pylori* colonies. PIP was also found to reduce levels of the inflammatory cytokine IL-1β and increase the anti-inflammatory IL-10 in *H. pylori*-stimulated cells. The microscopic examination of the gastric mucosa showed an inhibition of neutrophil and mononuclear cell infiltration in the antrum and corpus [27]. PIP has shown an inhibitory effect on the growth and adhesion of *H. pylori* to GC cells and reduces motility by suppressing the expression of the flhA gene, which encodes an integral component of the flagellar membrane, and the flgE gene, which encodes a flagellar hook component [28]. Another study showed that PIP also inhibits the translocation of *H. pylori* toxins (VacA) to GC cells and reduces the secretion of IL-8, high levels of which are found in *H. pylori* infection. In infection, bacteria enter the intercellular space through cleavage of the E-cadherin ectodomain, while β-catenin accumulates in the nucleus. It has been demonstrated that PIP both inhibits E-cadherin cleavages and reduces β-catenin expression, which could be used to prevent GC initiation [29].

In addition to its numerous therapeutic effects, another advantage of PIP is that it is a safe substance. This is confirmed by a 90-day study carried out on rats during which no significant adverse effects were demonstrated. However, due to the dose-dependent increase in cholesterol levels, 5 mg PIP/kg body weight/day is considered a safe dose [162]. PIP has also not shown genotoxicity [163]. The only toxic effect frequently indicated is adverse reproductive effects in males (impaired spermatogenesis) when piperine is used in bolus doses of 10 mg/kg body weight/day [164]. However, in a study of 60 days in mice administered PIP, it was shown that after a withdrawal period (120 days), the changes were reversible [165,166].

Studies have also shown that PIP interacts with a variety of drugs, leading to the improved bioavailability of test drugs, which may be associated with a risk of adverse drug reactions [164].

The clinical efficacy of PIP may be limited by its bioavailability due to its hydrophobic nature and poor water solubility [167]. A study in rats demonstrated that regardless of the route of administration of PIP (oral or intraperitoneal), approximately 97% was absorbed. Moreover, 3% of the administered dose was excreted in the feces, while no PIP could be detected in the urine. The study also demonstrates that PIP does not undergo any metabolic changes during absorption, as it was detected in both the serous fluid and the intestinal tissue of the rats tested. This was also confirmed by another study from 2007 [168,169].

### 2.8. Rosemary

Rosemary (*Rosmarinus officinalis*) is a popular spice native to the Mediterranean region. In addition to its use in cooking, rosemary has medicinal properties, including anticancer activity. The main constituents responsible for this property in rosemary are the diterpenes (carnosic acid, its derivative carnosol, and rosmarinic acid). Rosemary has been shown to be beneficial against colorectal cancer, pancreatic cancer, breast cancer, ovarian cancer, prostate cancer, and GC [170,171].

In a study using cell cultures of GC cell lines, crude rosemary fruit extract was proven to inhibit proliferation and induce the apoptosis of cancer cells. Rosemary stopped the cell cycle of GC cells at the G2/M stage, and this effect was greater when higher doses were used [172]. Another study tested the effect on GC of components of rosemary, particularly carnosol. Carnosol inhibits the growth of GC cells by inhibiting the autophosphorylation of RSK2 and the phosphorylation of its substrate, ATF1. The inhibition of RSK2 kinase activity occurred in a dose-dependent manner, with a greater dose. Carnosol also inhibited the cell cycle at the G2/M phase. In addition, an increase in the apoptosis of GC cells induced by carnosol was noted. A study was also conducted on mice, which were injected with GC tissues, and then one group was treated orally with carnosol. In this study, a reduction in the volume and weight of gastric tumors was observed, and treatment was given without significant weight loss. The expression of Ki-67 was investigated and was reduced, indicating an inhibition of proliferation [95].

Another compound in rosemary, sageon, has also been tested for its effect on GCs. It was found that sageon inhibits the growth and induces apoptosis of GC cells. It was also discovered that sageon downregulates the expression of Akt in GC cells, the increased phosphorylation of which affects the mechanism of resistance to cisplatin treatment. Therefore, sageon, by its action, could reverse the resulting resistance to cisplatin, which could be used in therapy [96].

Rosemary is considered to be a safe substance. In a study conducted on rats, no adverse effects, distressing symptoms, or mortality were observed. Furthermore, no changes were observed in biochemical tests, which demonstrates the low acute toxicity of rosemary extracts [173].

### 2.9. Galangal

The term galangal is used to describe the rhizomes of several varieties of this plant in the Zingiberaceae family [174]. It has its uses in cooking, traditional medicine, and cosmetics. Two varieties are most commonly described in the medical literature: Alpinia galanga and Alpinia officinarum [174,175,176,177]. Galangal is rich in phenolic compounds (acids and flavonoids), of which galangin is predominant. Other flavonoids include kaempferide, 3-methoxyl-galangin, kaempferol, and pinocembrin [178,179,180,181].

Galangin is a natural flavonoid compound [(3,5,7-trihydroxyflavone (C_15_H_10_O_5_)]. It exhibits anti-inflammatory, antimicrobial, antioxidant, antiviral, and apoptotic activities [30,31,32]. Research is being conducted on its use in the treatment of cancer, gastrointestinal diseases, diabetes, obesity, rheumatoid inflammation, arthritis, neuropathy, or osteoporosis [31,32].

It has been suggested that galangin may show promise in the treatment of GC. New studies indicate that galangin reduces the viability of the GC cell line MGC 803 by inducing early and late apoptosis, inhibiting cell proliferation, decreasing cell viability by modulating STAT3 activation, and increasing ROS production in vitro while showing low cytotoxicity to normal cells [31,97].

Cell apoptosis was associated with the decreased expression of Bcl-2 and caspase-3 (CASP3), increased protein expression of cleaved CASP3, and cleaved poly adenosine diphosphate-ribose polymerase (PARP) with no change in Bax expression in the GC cell line tested. It was observed that the substance caused a reduction in the expression of proliferating cell nuclear antigen (PCNA) and Ki67 mRNA and protein [31,97]. The effect of galangin on GC cell growth was also investigated in vivo by conducting a mouse study. A reduction in the ratio of p-JAK2/JAK2 (Janus-associated kinase) and p-STAT3/STAT3, protein expression of Bcl-2, CASP3, and Ki67 was demonstrated, while increased protein expression of cleaved CASP3 and cleaved PARP was observed [31,97]. A comparative study comparing the effects of galangin and quercetin on the SGC-7901 GC cell line showed greater efficacy of galangin, which inhibited cell growth, induced apoptosis, and reduced mitochondrial membrane potential (MMP). Apoptosis occurs through a mitochondrial pathway involving caspase-8/Bid/Bax activation [98]. Previous in vitro studies of human GC SNU-484 cells suggested that galangin slows their growth. The mechanism of action was related to morphological changes of the nucleus in the cells in question, where features of apoptosis associated with the decreased expression of Bcl-2 and Bcl-xl and the increased expression of the Bax protein were shown. Galangin increased the expression of caspase 3 and 9 (CASP9) and PARP polymerase while inhibiting ERK1/2 activity and stimulating c-Jun N-terminal kinases (JNK). It was also shown to increase the expression of ubiquitin carboxy-terminal hydrolase isozyme L1 (Uch-L1) while decreasing the expression of glutathione S-transferase P (GSTP), suggesting an antitumor effect of galangin by a particular mechanism [32]. One study showed that galangin likely inhibits the growth of MGC803 GC cells in vivo in nude mice and in vitro by suppressing the NF-κB pathway and enhancing autophagy. It was observed to inhibit cell viability, increase microtubule-associated protein 1 light chain 3 B (LC3 B), inhibit phosphorylation of proteins associated with the NF-κB pathway, and promote autophagosome formation in the cells tested [99]. An in vitro study on the human gastric adenocarcinoma (AGS) and L929 AGS cell lines showed that the aqueous extract prepared from the whole plant exhibited antiproliferative activity, especially when high concentrations of the preparation were used [182].

Based on studies conducted to date, it has been suggested that galangin produces a lesser cytotoxic effect compared to the 5-FU. Furthermore, studies in nude mice showed that, compared to 5-FU, galangin was not associated with significant weight loss [97].

No reliable studies on the bioavailability of galangin have been identified. It is suggested that further experiments are needed, and studies are being conducted to obtain extracts that are well absorbed from the gastrointestinal tract [176]. The use of galangin in clinical settings may be hampered by its characteristic chemical properties (as a 3-hydroxyflavone, it has low solubility in water) [31].

One study in rats reports that the substance can modulate the activity of cytochrome P450 (CYP) enzymes and, thus, may have the effect of improving the bioavailability of oral drugs [183]. The bioavailability of galangin is estimated to be 7.6% [175]. Other experiments conducted in rats indicate that the bioavailability of galangin is dependent on the route of administration of the substance. Oral administration had a lower bioavailability than intravenous administration. This is also confirmed by another study in mice where galangin was probably rapidly adsorbed and glucuronidated after oral administration [184].

*H. pylori* infection is associated with GC development [33]. The inhibitory effect of galangin on the secretion of IL-8 by human AGS cells infected with *H. pylori* and its antimicrobial activity has been demonstrated, confirming its anti-inflammatory effect [33,34].

An experiment was conducted to investigate the effect of galangin on its protective potential of GC against cancer development after benzoapirene induction. The results presented suggest that galangin administration prevented tumors in 37.5% of the animals. In the rest of the animals receiving galangin, significantly smaller tumors were observed, thus confirming the anticancer potential of the substance [35].

### 2.10. Coriander

Coriander (*Coriandrum sativum*) is a plant cultivated for its leaves and seeds, which are used in cooking, cosmetics, and medicine. There are reports of its anti-inflammatory and anticancer potential [185,186]. Coriander seeds contain phytochemicals such as geranyl acetate, linalool, and camphor. The anticancer potential is believed to be due to quercitin and linalool found in coriander [100,174,185,186,187,188].

Quercitin, which belongs to the flavonoids, occurs in various forms, the most common being glycosides and ethers: 3-O-glycoside of quercetin, quercetin 3-sulphate, quercetin 3-glucuronide, and quercetin 3′-methylether. The antioxidant, anti-inflammatory, antibacterial, and antiviral effects of the substance have been described. It also has its part to play in the treatment of cardiovascular diseases, cancer, metabolic disorders, and neurodegenerative diseases [189]. The exact molecular mechanisms of quercetin useful for GC therapy are being sought and require further research. It may interact through multiple biological pathways and processes [100,190,191].

It has been suggested that the substance has anticancer effects by inducing apoptosis, ferroptosis, necroptosis, and other forms of programmed cell death. In a study, quercetin was shown to have a concentration-dependent inhibitory effect on GC cell growth. The increased expression of pyroptosis proteins [Gasdermin D (GSDMD), Gasdermin E (GSDME), cleaved caspase-1 (CASP1), NLR family pyrin domain containing 3 (NLRP3)] and apoptosis markers (CASP3 and PARP) in quercitin-treated cells was also demonstrated [100]. The involvement of quercitin in the PI3K/Akt/P-gp (protein kinase B/P-glycoprotein) cascade was investigated in the oxaliplatin-resistant (OxR) GC cell line KATOIII/OxR. It has been shown to reduce tumor cell survival and contribute to oxaliplatin resistance (reduced P-gp expression and activity) [101]. Studies conducted on various GC cell lines using different doses of quercitin resulted in the following effects: the inhibition of proliferation, induction of apoptosis, inhibition of cell growth, and increased efficacy of chemotherapy with irinotecan/SN-38 (irinotecan/7-ethyl 10-hydroxy camptothecin) [102].

In an in vitro study conducted on the EPG85-257P cell line and its daunorubicin-resistant variant, EPG85-257RDB, quercetin was shown to have an antiproliferative effect on the cells tested. It is suggested that the substance may reduce the resistance of GC cells to daunorubicin [103]. In another study, quercetin was shown to be involved in the tumor necrosis factor (TNF) and IL-17 pathways, effectively targeting GC cells. It was found to inhibit GC cell division and promote apoptosis by affecting the tumor suppressor protein p53 gene (TP53), gene c-myc (MYC), and the tissue inhibitor of metalloproteinases (TIMP1) [104].

One study found that the use of quercitin was associated with reduced migration and the invasion of GC cells from the BGC823 and AGS lines. The reduced expression of urokinase plasminogen activator (uPA) and uPA receptor (uPAR) proteins was also observed [192]. A study conducted on GC stem cells attempted to establish the inhibitory effect of quercitin on their survival. The mitochondrial-dependent induction of apoptosis was observed (the activation of CASP3 and CASP9, downregulation of Bcl-2, and upregulation of Bax and cytochrome c (cyt-c)). The induction of apoptosis dependent on the blockade of PI3K-Akt signaling in gastric cancer stem cells (GCSCs) is a future target for gastric treatment [105].

Quercitin has low bioavailability and is poorly soluble in water. Research is being conducted into the use of quercitin glucosides, which are more bioavailable than the basic form. It is indicated that nano-quercitin has higher efficacy [189,191]. Another potential way to increase the bioavailability of quercetin is through cryopreservation and microencapsulation [193].

*Coriandrum sativum* was analyzed for the generation of ROS and IL-8 in *H. pylori*-infected AGS cells. The inhibitory effect of the extract in question on their production was demonstrated, which may suggest the usefulness of its use in gastrointestinal diseases, especially those associated with *H. pylori* [33].

### 2.11. Wasabi

Wasabi (*Eutrema japonicum*, *Wasabia japonica*) belongs to the Brassicaceae family and is a traditional spice used in cooking. In addition to its culinary value, wasabi is used in medicine due to its content of allyl isothiocyanate, flavonoids, phenylpropanoids, and carotenoids. Antiproliferative, anti-inflammatory, antioxidant, anticancer, neuroprotective, and antioxidant activities are indicated [194]. Of the substances contained in wasabi, two are being investigated for efficacy against GC cells; these are 6-(methylsulfinyl)hexyl isothiocyanate (6-MITC) and allyl isothiocyanate (AITC) [195,196,197].

AITC is formed by hydrolysis of glucosinolates from cruciferous vegetables, including wasabi [198]. Its inhibitory effect on AGS was already suggested in a study published in 1991 conducted on rats [64]. This study showed that the dietary administration of wasabi powder inhibited MNNG-induced AGS in rats [199].

A study in Taiwan showed that AITC can inhibit the migration and invasion of human AGS cells in vitro. The substance’s mechanism of action involved the inhibition of PI3K/Akt, uPA, and MAPK signaling pathways. Decreases in the levels and activity of matrix metalloproteinase-2 (MMP-2), matrix metalloproteinase-9 (MMP-9), and vimentin [106,107] were observed. AITC was shown to reduce the viability of AGS and SNU-1 GC cells in vitro. The mechanism of action of AITC was to induce morphological changes in tumor cells [108].

AITC extracted from the leaves of wasabia japonica, when administered orally to *H.pylori*-infected gerbils, resulted in a reduction in symptoms and had no effect on body weight or gastric pH [36].

AITC is non-toxic and safe. It is used as an additive in food products [106].

The administration of a substance containing 6-MITC in high doses to healthy men has not resulted in any side effects [200]. AITC has a high bioavailability, with approximately 90% of the product being absorbed when taken orally [106]. To improve the solubility and stability of AITC, nano- and microemulsions are used, improving its antitumor activity [198].

Sulforaphane (SFN), present in wasabi, may also exhibit anticancer properties [201]. SFN is one of the isothiocyanates whose efficacy in the treatment of GC has been confirmed in vitro and in vivo in nude mice. Its action resulted in the inhibition of cancer cell activity through the induction of PDL-1 expression in diseased cells [109]. The effects of sulforaphane on glycolysis and proliferation of SGC7901 and BGC823 GC cells were also addressed, taking into account the role of the TBX15/KIF2C axis (T-box transcription factor 15/kinesin superfamily member 2C). The substance was found to inhibit GC cell proliferation by inhibiting glycolysis, which is mediated by the downregulation of pyruvate kinase muscle isozyme M2 (PKM2). In addition, the overexpression of TBX15 results in the inhibition of glycolysis and tumor cell proliferation [110]. The mechanisms of action of sulforaphane on nicotine-stimulated MMP-9 induction have been investigated, and the substance has been shown to inhibit MMP-9 expression by reducing ROS production, inhibiting p38 MAPK and ERK1/2 activation, which, in turn, inhibits Activating protein 1 (AP-1) and NF-κB activity [202]. It has been shown that SFN can inhibit GC cell autophagy through the activation of the miR-4521/PIK3R3 (phosphoinositide-3-kinase regulatory subunit 3) pathway. The mediator miR-4521-dependent apoptosis in vitro was also observed [111]. Sulforaphane has been shown to have proliferation inhibitory, cell cycle arrest, and apoptosis induction abilities in GC cells. The suppression of SET and MYN-domain containing 3 (SMYD3) associated with the regulation of cysteine-rich angiogenic inducer 61 (CYR61) and myosin regulatory light polypeptide 9 (MYL9) was observed. SMYD plays an important role in the anticancer effects of SFN [203].

### 2.12. Cinnamon

Cinnamon is extracted from the inner bark of the cinnamon tree. The active compounds of cinnamon are eugenol, cinnamaldehyde, beta-caryophyllene, and beta-caryophyllene oxide [204].

Eugenol has an inhibitory effect on AGS cell proliferation. It is a strong oxidant and has a cytotoxic effect on cancer cells, being safe for non-cancerous cells [44]. Eugenol has anti-metastatic activities on AGS cell lines independent of p53, P21, and SMAD4. It also inhibits the secretion of the TGF-β type 2 isoform and the intracellular expression of TGF-β. Compared to capsaicin, eugenol is a weaker antiproliferative agent; however, it is more effective in SMAD4 null SW620 cells and the presence of TGF-β receptor inhibitor LY2109761 in the SW620 cell line [120]. Manikandan et al. [112] proved the antiproliferative effect of eugenol for nuclear factor-kappaB (NF-κB) family members ((NF-κB (p50 and p65), an inhibitor of kappa B alpha (IκBα), phosphorylated IκBα (p-IκBα), IκB kinase β (IKKβ)) and the NF-κB target genes that cause proliferation and cell survival, including those that promote (e.g., cyclins, PCNA) or inhibit (e.g., p53, p21, and Gadd45). The activation of NF-κB in N-methyl-N(‘)-nitro-N-nitrosoguanidine-induced gastric tumors correlated with increased levels of cyclins and PCNA while simultaneously reducing the expression of p53 and p21. In the mentioned study, the administration of eugenol to animals treated with MNNG changed this ratio, i.e., there was a decrease in substances responsible for cell cycle promotion and an increase in those responsible for cell cycle inhibition. This allows for limiting tumor growth in the process of carcinogenesis [112]. To enhance the activity of eugenol, other scientists created a range of eugenol β-amino alcohol and β-alkoxy alcohol derivatives, which were subsequently examined against cancer cell lines, specifically AGS. The results indicated that certain derivatives have stronger cytotoxic properties compared to eugenol, leading to a statistically significant decrease in cell viability. Neither of the molecules led to a decrease in viability in noncancerous cells, indicating their selectivity towards cancer cells. The researchers explained that the cytotoxic properties of the compounds were linked to the induction of apoptosis, as they activated caspases-3, -8, and -9 [121]. More importantly, the mutation of p53 is frequently observed in human cancers, leading to malfunctions in apoptosis and rendering cancer cells resistant to chemotherapy, such as in GC. The responsiveness of GC to chemotherapy has been demonstrated to diminish when p53 is absent. A Western blot analysis of pro-apoptotic markers showed that eugenol could stimulate caspase-8 and caspase-3 even when p53 was absent [113].

Cinnamaldehyde (CA) induces antiproliferative effects by reducing cell viability in a dose-dependent manner compared to the control across different types of GC cells. The findings revealed that CA induces endoplasmic reticulum stress and autophagic cell death by activating the PERK-CHOP signaling pathway, inhibiting G9a binding on the Beclin-1 and LC3B promoter, and disrupting the Bcl-2–Beclin-1 interaction in GC cells [114].

### 2.13. Oregano

*Origanum vulgare*, known as oregano, is a widely used aromatic plant.

Sri Renukadevi Balusamy et al. [115] evaluated oregano essential oil apoptotic effects against human stomach cancer cell lines. They extracted four major components from oregano oil: thymol, ρ-cymene, γ-terpinene, and carvacrol. When the cells were treated with different concentrations of the tested spice, reduced cell numbers were recorded, so this scientific report proves the potential antiproliferative effect of oregano essential oil (EO) in a dose-dependent manner. EO extracted from this plant also caused a decrease in the ability to migrate human GC cells, while the control cells had not lost this possibility. Cancer cells can grow more easily than healthy cells; therefore, they need more energy than healthy cells, which is obtained during the activation of de novo fatty acid synthesis. To evaluate the molecular mechanism involved in cancer cell death, they investigated genes involved in the fatty acid and cholesterol biosynthesis pathway, specifically 3-hydroxy-3-methylglutaryl-coenzyme A reductase (HMGCR), Acetyl CoA synthase (ACC), sterol regulatory element-binding protein (SPREPB1), and fatty acid synthase (FASN). The expression of pathway genes like HMGCR, ACC, SPREPB1, and FASN showed a decrease in both transcript profile and protein accumulation, leading to the inhibition of GC cell growth. Moreover, oregano EO triggered the activation of Bax (a pro-apoptotic protein), reduced the levels of BCL2 (anti-apoptotic proteins), and, consequently, induced apoptosis via the mitochondrial pathway [115].

Another study initiated by Ayse Günes-Bayir [53] showed the cytotoxic, genotoxic, apoptotic, and ROS-generating effects of carvacrol (5-isopropyl-2-methylphenol) against the human AGS in the in vitro system. There was a positive correlation observed between the increasing dosage of carvacrol and the relative ROS levels in GC cells, leading to a pro-oxidant condition and induction of apoptosis thereafter. Moreover, Western blot analyses of carvacrol-treated cells suggested that the tested compound induces apoptosis, decreasing the Bcl-2/Bax ratio [53]. Carvacrol may become a new anticancer compound, but it is not selective towards pathological cells, as investigators proved by comparing its effects on GC cells to those on normal human fibroblast [205]. A recent study performed by the above-mentioned author investigated the clinical significance of this compound in a normal stomach, this time in an in vivo system. Low doses of carvacrol (10 and 25 mg/kg body weight) can prevent the toxic effects of MNNG by reducing the expression of caspase-9 and Bax and the high expression of Bcl-2 protein levels. The expression levels of caspase-9 and Bax proteins were notably elevated in the group with high-dose carvacrol (100 mg/kg BW carvacrol and MNNG) in comparison to all other groups. This observation might indicate the potential apoptotic activity of carvacrol at high doses. However, a biochemical analysis was performed to determine which dose of carvacrol provided the best protection against GC. The results of serum and tissue analyses of cytokines (IL-1β, IL-6, and TNF-α) and other investigated inflammatory mediators (VEGF and TGF-β) suggest that carvacrol can significantly reduce inflammation in low doses (10 mg/kg BW). We know that a large number of reports have shown that systematic inflammation plays a significant role in carcinogenesis, so this result is valuable to the scientific world. The authors reported that carvacrol at low doses showed a significant chemopreventive effect against GC in rats [37].

With the increased use of two compounds, carvacrol and thymol, in the food industry, people may be increasingly exposed to them orally. Lots of studies have focused on their beneficial effects, but according to the researchers, they may also have toxic action. The digestive tract, especially the stomach, would be the first target of these agents consumed by humans. Investigators indicated that these compounds should be used carefully in the food industry; therefore, further studies on their safety for human health are needed [206]. Recently, it was documented that higher doses of the phenolic compound, which is thymol, may equally harm cancerous and healthy cells, but administering low doses seems to protect healthy cells without losing its anticancer effect [207].

### 2.14. Cardamom

Cardamom belongs to the Zingiberaceae family, like turmeric and ginger. His main bioactive compounds are as follows: 1,8-cineole, α-terpinyl acetate, nerolidol, sabinene, g-terpinene, α-pinene, methyl linoleate, α-terpineol, β-pinene, n-hexadecanoic acid, and limonene [208].

Manjunath et al. [209] studied cardamom oil, lemon oil, and jasmine oil for their cytotoxic activity against human skin, gastric, and brain cancer cell lines. Unfortunately, cardamom oil (Elettaria cardamomum) showed stronger cytotoxicity against skin cancer cell lines only, but it did not show an inhibition of stomach cancer cell growth [209]. Another study initiated by Samir Qiblawi et al. [38] showed the protective effect of cardamom against forestomach chemical carcinogenesis. This study investigated the chemopreventive potential of cardamom against benzo(a)pyrene [B(a)P]-induced forestomach papillomagenesis in mice. The results showed that treatment with cardamom [(B(a)P + cardamom)] significantly reduced tumor incidence and multiplicity by 41.67% and 74.55%, respectively, compared with the B(a)P control group. Because cardamom was administered before the onset of cancer, its effect can be considered to be preventive on the development of B(a)P-induced forestomach papillomagenesis. The potential chemopreventive properties of cardamom may be ascribed to its ability to modulate phase II detoxifying enzymes, particularly glutathione-S-transferases (GST); activate antioxidant enzymes; elevate GSH levels; and inhibit lipid peroxidation levels as well as LDH activity. No adverse effects on ingestion of cardamom were detected. In conclusion, it seems reasonable to consume cardamom as a preventive agent against stomach cancer [38].

### 2.15. Fenugreek

Fenugreek seeds contain simple alkaloids, which consist mainly of trigonelline, choline, gentianine, and carpain. Other constituents include saponins, steroidal sapogenins, and flavonoids such as quercetin, luteolin, vitexin cinnamate, vicenin, and isovitexin, which are believed to support the anticancer effects of fenugreek [210].

Diosgenin, a major sapogenin found in fenugreek seed, has shown high potential and interest in the treatment of various cancers such as GC [211]. Previous studies have shown that diosgenin has effects that inhibit mesoderm posterior 1 (MESP1) expression, leading to suppressed proliferation of GC cells via inducing alternative reading frame (ARF) expression. MESP1 belongs to the family of transcription factors and promotes the proliferation of GC cells by inhibiting the expression of ARF, which is a tumor suppressor in human cancer. Therefore, diosgenin could be a potential natural product for the treatment of GC [116]. Another hallmark involved in carcinogenesis is invasion and metastasis. Interestingly, Mao et al. [117] showed that diosgenin could have an anti-invasion effect on GC cells because it may significantly enhance the expression of cell adhesion molecules, including E-cadherin, an invasion suppressor molecule. In addition, when combined with HIF-1α-specific short hairpin RNA (shRNA), diosgenin can inhibit cells more effectively. These findings indicate that diosgenin could be an effective compound in managing GC cells in hypoxia conditions [117]. More recent evidence shows that treatment with diosgenin alone causes a dose-dependent decrease in the cell viability and induces significant increases in G0/G1 cell cycle arrest and apoptosis. However, combined treatment often produces a stronger suppressive effect on tumor cellular function compared to therapeutic outcomes achieved with monotherapy. This was proven in an experiment using diosgenin and GSK126 on GC cells because they synergistically may induce even stronger effects on impaired cell proliferation [118].

### 2.16. Caraway

*Carum carvi* (caraway) is a plant of the Apiaceae family. In folk medicine, it is used for indigestion, galactagogue, pneumonia, or eczema [39]. The main components of caraway fruit essential oil are d-carvone, limonene, and myrcene. The percentage of these substances in the oil varies depending on the ecotype and the prevailing environmental conditions [40,212].

D-carvone provides various biological effects, such as antiproliferative, antioxidant, antidiabetic, anti-inflammatory, anti-convulsant antimicrobial, fungicidal, antidiabetic, and insecticidal properties [40,41,42]. Studies on the effects of d-carvone on stomach cancer have shown its ability to inhibit cell proliferation, increase ROS production, and induce apoptosis. It induces a dose-dependent loss of mitochondrial membrane potential. In cells treated with d-carvone at a dose of 20 µM, there was loss of cell structure and chromatin condensation, while at a dose of 25 µM, there was damage to nuclei and complete cell rupture [4]. In addition, d-Carvone downregulates the JAK/STAT2 signaling pathway in GC cells and inhibits JAK/STAT3 signaling pathway in GC cells. However, further research is needed to understand the exact role of d-carvone [41].

Another study assessing the impact of caraway (*Carum carvi*) extracts on the 2, 3, 7, 8-tetrachloro-dibenzo-p-dioxin-dependent gene expression of cytochrome P450 1A1 in the rat H4IIE cells showed its inhibitory effect. At concentrations above 0.13 µM, there was inhibition of EROD activity; higher concentrations (1.3 and 13 µM) caused an approximately 10-fold suppression of enzyme activity. These substances have the potential to reverse TCDD-dependent induction of cytochrome P450 1A1 and inhibit chemo-induced tumor growth, but there is a need for further research in this area [213].

D-limonene presents antioxidant, anti-inflammatory, and anticancer effects. In a study conducted on nude mice with human GC implanted as a result of the use of d-limonene, a decrease in tumor weight and a decrease in the incidence of liver and peritoneal metastases have been observed mainly due to the induction of apoptosis by limonene [119]. In another study on the synergistic effect of limonene with berberine, limonene shows cytotoxic effects in cells in the MGC803, induces apoptosis, has antioxidant effects, and reduces mitochondrial membrane potential (MMP); lower Blc-2 expression increases caspase-3 expression. This effect is stronger when d-limonene with berberine is used simultaneously than when these substances are used separately. Further studies are needed to determine the mechanism of the synergistic effect of the two substances [214].

Nonspecific lipid transfer proteins (nsLTPs) are found in different plants, but due to their large number, their exact role is not known. They participate in the detection, presentation, and modification of lipids and post-translational transfer between membranes in the cytoplasm. Studies on the nsLTPs1 protein extracted from caraway seeds showed its antioxidant, antiproliferative, and inhibitory effects in a dose-dependent manner against MDA-MB231 and MCF-7 breast cancer cells. However, further studies evaluating the effect of cumin nsLTPs1 proteins and obtaining confirmation of the antiproliferative effect in vivo are needed [39].

Caraway in therapeutic doses is well tolerated and shows no toxic effects. Caraway oil is not recommended for patients with liver disease, gallstones, and other biliary diseases (caraway has an inhibitory effect on gallbladder emptying), achlorhydria, and nephritis. Due to limited data, caraway oil is also not recommended for use during pregnancy and lactation [40].

### 2.17. Clove

*Syzygium alternifolium* (SA) is part of the Myrtaceaeu family. It is found geographically in the southeastern Ghats [215]. *Syzygium alternifolium* has various medicinal uses. Its fruits are used to treat stomach aches and ulcers and treat rheumatic pain; its seeds are used as anti-diabetic agents; its leaves are used to treat dry cough and dysentery; and its stem bark is used as an antiseptic. In addition, SA has hypoglycemic and antihyperglycemic effects [45,215].

The main component of clove oil is eugenol. This oil also contains flavonoids, eugenol acetate, β-caryophyllene, α-humulene, and gallic acid [45,121,216].

Babu TM et al. [45] evaluated the efficacy of three flavonoids in inhibiting HER2 on AGS cell lines. They were (1) 5-hydroxy-7,4′-dimethoxy-6,8-di-C-methylflavone (eucalyptin), (2) kaempferol 3-*O*-*β*-d-glucopyranoside, and (3) kaempferol 3-*O*-α-l-rhamnopyranoside. This study showed that compounds 1, 2, and 3 isolated significantly inhibited the proliferation of human GC cells by arresting the G2/M phase of the cell cycle. Due to the significant activity in the in silico approach in terms of binding affinity to HER2 of substance 2, three new flavonoid analogs for compound 2 from the ZINC database were also checked, namely ZINC67903192, ZINC59763389, and ZINC85816423. These substances have more H-bond acceptor/donor characteristics than compound 2, which improved specificity and selectivity with high binding affinity toward HER2. These studies point to the possibility of developing HER2 inhibitors from natural sources in the future [45].

Clove’s preventive effect on GC is related, among other things, to its antibacterial activity against *H. pylori*. In addition to its antibacterial activity, eugenol essential oil (EEO) exhibits anti-inflammatory effects. There was no development of resistance against EEO, and it showed activity against biofilm at concentrations of 25 µg/mL and 50 µg/mL against various strains of *H. pylori*, with suppression percentages of 49.32% and 73.21%, respectively [46]. The braking zone varied according to the study, ranging from 10 ± 06 to 22 ± 04 mm [46,47,48].

Oral rinses with clove extract have a bacteriostatic effect on *H. pylori*. This action promotes a reduction in the spread of the pathogen and the recurrence of the infection. The efficacy varies depending on the bacterial strain (highest efficacy against slow-growing strains). In addition to growth inhibition, a decrease in CagA expression in *H. pylori* was also achieved. A limitation may be the risk of potential allergenic effects of rinses containing essential oils [217].

Various animal studies indicate that eugenol has gastroprotective effects. It reduced the severity of lesions and ulcers caused by ethanol and increased the production of gastric mucus without affecting gastric secretion [218,219]. Another study found that eugenol treatment of ethanol-induced ulcers decreased plasma levels of NO, TNF-α, and IL-6 and increased PGE2. Additionally, eugenol showed significant transcriptional and translational upregulations of HSP70 and a downregulation of iNOS in rat gastric tissue [220]. Eugenol has poor bioavailability due to its lipophilic nature. To increase its bioavailability, it is used eugenol nano-formulations like liposomes, nanoparticles, microemulsions, and micelles [221]. It is not toxic. The maximum tolerated dose for humans is 1024 (mg/kg/day). It is well absorbed in the gastrointestinal tract and is almost completely excreted in the urine. Due to its short half-life and rapid metabolism, there is a malevolent risk of accumulation [222].

### 2.18. Dill

*Anethum graveolens* (dill) is an herb of the family Apiaceae, which is widely used for both culinary and medicinal purposes. It is used to relieve various ailments such as digestive disorders, soothe stomach irritation, lower lipid and glucose levels, and stimulate lactation. In addition, it exhibits anti-inflammatory, antioxidant, anticancer, and antimicrobial effects [223].

Dill is rich in a variety of substances, such as phenols, flavonoids, tannins, spronines, terpenes, and cardiac glycosides. The main constituents of dill seed essential oil are carvone and d-limonene [49]. There is a lack of current research evaluating the effect of dill extracts on the treatment of GC. Available studies describe its antioxidant and antiradical, anti-glycation effects [50,51]. In a study on mice, *A. graveolens* seed extracts show effective antisecretory and anti-ulcer effects against HCl and ethanol-induced gastric lesions. This effect may be related to the presence of terpenes and flavonoids; however, further research is needed to determine the exact mechanism of action of dill seeds [52].

Dill can be used in the prevention of GC due to its antimicrobial activity against *H. pylori*, an infection which is a risk factor for the development of GC. Thanks to its bacteriostatic effect, it can complement currently used therapies [224].

Dill is “generally recognized as safe” (GRAS) as a food by the U.S. Food and Drug Administration. It is generally well tolerated, although occasional allergic skin reactions have been reported, especially after contact with fresh dill. In two studies, nursing mothers were administered d-carvone. No adverse effects were observed in mothers or infants [225]. *A. graveolens* compounds show good gastrointestinal absorption, lipophilicity, and bioavailability (0.55–0.58). α-pinene, β-pinene, and limonene inhibit four isoenzymes of cytochrome P450 (CYP2C9), while ρ-cymene and meta-cymene inhibit four isoenzymes of cytochrome P450 (CYP2D6) [226].

### 2.19. Thyme

The Thymus genus, which belongs to the Lamiaceae family, has 350 species, including *T. vulgaris*, *T. serpyllum*, *T. capitatus*, *T. sipyleus*, and *T. schimperi* [227]. Thymus vulgaris is a source of many biologically active compounds such as phenols, tannins, glycosides, and flavonoids. The main acts of *T. vulgaris* are thymol, p-cymene, eugenol, and γ-terpinene. Other compounds present in thyme are carvacolic acid, chlorogenic acid, caffeic acid, benzoic acid, synapinic acid, gallic acid, kemferol, myricetin, and quercetin [53,54].

Thymol exhibits genotoxic and cytotoxic effects on AGS cells in a dose-dependent manner [54,55]. It induces apoptosis by producing ROS (more than a three-fold increase) in various cancer cells and regulates the cell cycle by prolonging the sub-G1 cellular phase in AGS cells. In addition, thymol damages MMPs and activates proapoptotic proteins, Bax, PARP, and caspase-7, -8, and -9. No significant changes were observed in the expression of Bcl-2 [54]. Another study found a decrease in Bcl-2 in a dose-dependent manner and an additional increase in caspase-3 [55]. Another compound contained in thyme is chlorogenic acid, which shows an affinity for GC target genes. In addition, it shows strong anticancer activity against various cancer and non-cancer cell lines (it is active against embryonic lung fibroblasts). It meets all five Lipinski rules and has good lipophilicity and water solubility characteristics [53].

Swiss ADME analysis showed that thymol, carvacrol, p-cymene, and eugenol had good water solubility. These compounds showed high absorption in the gastrointestinal tract with low glycoprotein permeability and no inhibitory effect on CYP2C19 and CYP2C9. The results showed that ethyl acetate and n-butanol fractions of T. vulgaris were safe for oral administration at 800 mL/kg body weight and showed no signs of toxicity. In rats with ethyl acetate- and n-butanol-induced hepatotoxicity, slight decreases in ALT, AST, and ALP activities were observed. If present, ROS-induced hepatotoxicity can be effectively controlled by administering agents that exhibit antioxidant, free radical scavenging, and lipid antioxidant activity [53]. In the case of thymol, there are reports of induction of chromosome number abnormalities when used at the highest concentrations. In vivo studies are needed to confirm whether lower doses will be better tolerated by healthy cells while maintaining efficacy against cancer cells [55].

### 2.20. Piper Sarmentosum

*Piper sarmentosum* is an herb about 20 cm tall found in India, Malaysia, Thailand, as well as China. It is a traditional medicinal plant used to treat cough, fever, stomachache, and toothache. Modern research shows that PS has antibacterial, insecticidal, hypoglycemic, anticancer, and hypotensive effects. The main constituents are volatile oil and alkaloids. In addition, it contains small amounts of sterols and lignans [228].

The study from 2023 tested the protective effect of methanolic extract of *Piper sarmentosum* on gastric mucosal damage as an alternative to other antioxidants. On a group of test rats, *Piper sarmentosum* supplementation significantly reduced the results of gastric lesions and, interestingly, proved more effective than omeprazole. This study shows that oral *Piper sarmentosum* supplementation supports protection against the occurrence of gastric lesions, so it is a potential therapeutic agent for gastric ulcers [56]. Unfortunately, studies to date do not describe a direct effect of *Piper sarmentosum* on gastric cancer. In addition to those mentioned above, additional effects of *Piper sarmentosum* have been noted. These include promoting fracture healing, neuroprotective, antidepressant, and anti-atherosclerotic effects. However, conclusive data on its safety are lacking [228].

### 2.21. Basil

Basil is a medicinal herb native to subtropical regions of Asia, Africa, and Central and South America. Essential oils of basil include linalool, estragole, methyl cinnamate, 1,8-cyneol, methylchavicol, eugenol, bergamotene, α-cardinol, limonene, geraniol, and camphor. The anticancer activity of basil extract has been proven [122].

Anticancer effects of basil have been described based on mechanisms such as cell death and the inhibition of cell viability, cytotoxicity, antioxidant activity, apoptosis, reduced tumor growth, and cell cycle arrest. In the case of human GC, *Ocimum basilicum* leaf extract proved less toxic to tumor cells than Impatiens walleriana, which was tested at the same time. However, cytotoxic effects did occur. This can be attributed to the anthocyanin and flavonoid derivatives present in the extracts [122].

## 3. Conclusions

Commonly used herbs and spices contain substances with antineoplastic properties (e.g., saffron, black pepper, rosemary, cinnamon). Many of them (e.g., turmeric, ginger, garlic, black pepper, galangal, wasabi) also show anti-inflammatory and immunomodulatory effects. Further, some spices (black cumin, coriander, cardamon) can influence important risk factors for GC, such as *H. pylori* infection. The effect of chili peppers and other capsaicin-containing plants on GC remains controversial. Future research should focus on the use of not only spices and herbs in the prevention and therapy of GC but also the direct use of compounds in these plants.

## Figures and Tables

**Table 1:** Potential use the spices for the prevention of GC.

Spice	Active Compounds	Use in GC Prevention	Additional Information
Turmeric [16,17,18,19]	curcumin	exhibits anti-inflammatory, antioxidant properties; antibacterial properties are related to the inhibition of *H. pylori* infection;	-
Ginger [20,21,22]	6-gingerol 8-paradol	shows anti-inflammatory effects and inhibition of *H. pylori* infection;	-
Garlic [23,24]	allicin	lack of strong evidence regarding the use of garlic in the prevention of GC	-
Black cumin [25,26]	thymoquinone	shows activity against *H. pylori* infection	combination of black cumin with honey (12 also shows activity against *H. pylori*
Black pepper [27,28,29]	piperine	inhibition of gastritis caused by *H. pylori*, reduction of the number of *H. pylori* colonies, inhibition of *H. pylori* adhesion to GC cells and reduction of their motility, inhibition of the translocation of *H. pylori* toxins	-
Galangal [30,31,32,33,34,35]	galangin	exhibits anti-inflammatory, antioxidant, antimicrobial effects (inhibitory effect on *H. pylori*),	anticancer potential of galangin by inhibiting benzoapirene-induced gastric cancer development
Coriander [33]			inhibitory effect on ROS and IL8 generation of coriander extract on *H. pylori*-infected AGS cells,
Wasabi [36]	allyl isothiocyanate	reduction of symptoms associated with *H. pylori* infection	-
Oregano [37]	thymol ρ-cymene γ-terpinene carvacrol	exhibits anti-inflammatory properties	reduction of expression cytokines (IL-1β, IL-6, and TNF-α), and other inflammatory mediators (VEGF and TGF-β)
Cardamon [38]	1,8-cineole, α-terpinyl acetate, nerolidol, sabinene, g-terpinene, α-pinene, methyl linoleate, α-terpineol, β-pinene, n-hexadecanoic acid, and limonene	reduction of tumor incidence and multiplicity	cardamon modulates phase II detoxifying enzymes, particularly GST, activates antioxidant enzymes, elevates GSH levels, and inhibits lipid peroxidation levels and LDH activity
Caraway [4,39,40,41,42]	d-carvone, limonene, nsLTPs	exhibits antioxidant, anti-inflammatory, and anticancer effects, inhibit cell proliferation, increase ROS production, and induce apoptosis	-
Clove [43,44,45,46,47,48]	eugenol, 5-hydroxy-7,4′-dimethoxy-6,8-di-C-methylflavone (eucalyptin), kaempferol 3-O-β-d-glucopyranoside, kaempferol 3-O-α-l-rhamnopyranoside	exhibits inhibitory effect on AGS gastric cancer cell proliferation, oxidant and cytotoxic effect on cancer cells, has antibacterial activity against *H. pylori*	-
Dill [49,50,51,52]	carvone, d-limonene	antioxidant, anti-radicals, antisecretory and anti-ulcer effects, antimicrobial activity against *H. pylori*	-
Thyme [53,54,55]	thymol, p-cymene, eugenol, carvacolic acid, chlorogenic acid,	exhibits genotoxic and cytotoxic effects on AGS cells, induces apoptosis in human AGS cells	-
*Piper sarmentosum* [56]	volatile oil, alkaloids, sterols, and lignans	*Piper sarmentosum* supplementation significantly reduced the results of gastric lesions. Oral *Piper sarmentosum* supplementation supports protection against the occurrence of gastric lesions	-

**Table 2:** Potential use the spices for the treatment of GC.

Spice	Active Compounds	Use in GC Treatment	Mechanism	Additional Information
Tumeric [57,58,59,60,61,62,63]	curcumin	inhibits proliferation, migration, inducts apoptosis	suppression of the Shh, Wnt, PI3K signaling pathways; activation of the P53 signaling pathway; epigenetic modification	epigenetic modifications involve upregulation of histone acetylation and deacetylation enzymes, upregulation of mir34, mir33b, downregulation of mir21 expression
Ginger [64,65,66,67]	6-gingerol 8-paradol	inhibits GC cell proliferation; induces apoptosis; increases the radiosensitivity of GC cells; increases the sensitivity of GC cells to cisplatin	8-Paradol induces tumor cell apoptosis by promoting PINK1/Parkin-mediated mitophagy of cells	-
Garlic [68,69,70,71,72,73,74,75,76,77,78,79]	allicin diallyl disulfide diallyl trisulfide s-allilocysteine	induces apoptosis, inhibits proliferation, and arrests GC cells in the G2/M phase of the cell cycle	allicin induces cytochrome release from mitochondria, hydroxylation of caspases, activation of p38 MAPK/caspase 3 pathway; diallyl disulfide is associated with the arrest of GC cells in the G2/M phase of the cell cycle; diallyl trisulfide activates AMPK	Garlic supplementation appears to have a beneficial effect on reducing the risk of death from GC; s-allilocysteine inhibits the activation of inflammatory mediators, which can be used to treat gastric ulcers
Black cumin [80,81,82,83]	thymoquinone	inhibits proliferation and induces apoptosis	inhibition of PI3K/Akt/mTOR and STAT3 pathway; potentiation the effects of 5-fluorouracil	-
Chili pepper [84,85,86,87,88]	capsaicin	inhibits proliferation and induces apoptosis of GC cells	increases production of caspase-3; reduces the expression of Bcl-2; reduces the expression of phosphorylated ERK 1/2, p38 MAPK, or JNK epigenetic modifications: restoring the activity of hMOF HATs	may increase the risk of GC
Saffron [89,90,91,92]	crocin crocetin	inhibition of GC cell proliferation; stimulation of apoptosis stimulation of apoptosis inhibition of angiogenesis	reduction of expression TPM4 increase in Bax/Bcl-2 ratio and activation of caspases reduction of the Bcl-2/Bax ratio inhibition of the sonic hedgehog signaling pathway	-
Black pepper [93,94]	piperine	inhibition of GC cell proliferation and induction of apoptosis inhibition of IL-6	inhibition of the PI3K/Akt signaling pathway suppression of p38 MAPK and STAT3	-
Rosemary [95,96]	carnosol sageon	induction of apoptosis, inhibition of the growth of GC cells, reduction in the volume and weight of the gastric tumor induction of apoptosis	inhibition of the RSK/CREB signaling pathway loss of the MMP and activation of caspase proteins	-
Galangal [31,32,97,98,99]	galangin	Induction of apoptosis inhibiting cell growth decreasing cell viability	decreased expression of Bcl-2 and CASP3, increased protein expression of cleaved CASP3 and cleaved PARP, reduced expression of PCNA and Ki67 mitochondrial pathway involving CASP8/Bid/Bax activation decreased expression of Bcl-2 and Bcl-xl, increased expression of Bax protein increased expression of CASP3, CASP9, and PARP polymerase, inhibition of ERK1/2 activity and stimulation of c-JNK reduction in the ratio of p-JAK2/JAK2 and p-STAT3/STAT3 and protein expression of Bcl-2, CASP3, and Ki67 increased protein expression of cleaved CASP3 and cleaved PARP suppression of NF-κB pathway and enhancement of autophagy modulation of STAT3 activation and increase in ROS	Increased expression of Uch-L1 while decreased expression of GSTP can suggest an antitumor effect of galangin by a particular mechanism
Coriander [100,101,102,103,104,105]	quercetin	inhibiting cell growth induction of apoptosis	increased expression of pyroptosis proteins: GSDMD, GSDME, CASP1, NLRP3, and apoptosis markers CASP3 and PARP Affecting TP53, MYC, and TIMP1 CASP3 and CASP9 activation, Bcl-2 downregulation Bax, and cyt-c upregulation	Quercetin is suggested to have a positive effect on reducing the degree of resistance of gastric cancer cell lines to daunorubicin (EPG85-257RDB) or oxaliplatin (KATOIII/OxR) and increased efficacy of chemotherapy with irinotecan/SN-38
Wasabi [106,107,108,109,110,111]	allyl isothiocyanate sulforaphane	inhibition of cell migration and invasion decreasing cell viability inhibition of cancer cell activity inhibition of cancer cell proliferation induction of apoptosis	inhibition of PI3K/AKT, uPA, and MAPK signaling pathways; decreased MMP-2 and MMP-9 activity changes in the expression of DNA damage and repair proteins PDL-1 induction glycolysis inhibition involvement of miR-4521-dependent mediator	-
Cinnamon [112,113,114]	eugenol cinnamaldehyde beta-caryophyllene beta-caryophyllene oxide	reduction of tumor growth, inhibition of GC cell proliferation induction of apoptosis inhibits the proliferation of GC cells and induces endoplasmic reticulum stress and autophagic cell death	antiproliferative effect of eugenol for NF-κB family members and the NF-κB target genes eugenol stimulates the expression of caspase-8 and caspase-3 cinnamaldehyde activates the PERK-CHOP signaling pathway, inhibits G9a binding on the Beclin-1 and LC3B promoter, and disrupts the Bcl-2–Beclin-1 interaction	decrease in substances responsible for cell cycle promotion and an increase in those responsible for cell cycle inhibition eugenol can stimulate caspase-8 and caspase-3 even when p53 is absent
Oregano [37,53,115]	thymol carvacrol ρ-cymene γ-terpinene	inhibition of GC cell proliferation and migration (oregano oil) decrease in protein accumulation involved in the fatty acid and cholesterol biosynthesis pathway (oregano oil) carvacrol induces apoptosis via the mitochondrial pathway carvacrol exhibits pro-oxidant properties	reduction of expression HMGCR, ACC, SPREPB1, FASN reduction of the Bcl-2/Bax ratio and activation of caspase 9 ROS-generating effect	potential apoptotic activity of carvacrol at high doses
Fenugreek [116,117,118]	steroidal sapogenins e.g., diosgenin, trigonelline, choline, gentianine and carpain, quercetin, luteolin, vitexin cinnamate, vicenin, and isovitexin, saponins	diosgenin: inhibition of GC cell proliferation inhibition of GC invasion decrease in the cell viability arrest of GC cells in the G0/G1 phase of the cell cycle induce apoptosis	reduction expression of MESP1, induction expression of ARF stimulation of expression of cell adhesion molecules, e.g., E-cadherin	
Caraway [4,41,119]	d-carvone limonene	inhibits cell proliferation, increases ROS production, and induces apoptosis and loss of mitochondrial membrane potential In a study on nude mice with human gastric cancer implanted, d-limonene, a decrease in tumor weight and a decrease in the incidence of liver and peritoneal metastases were seen	downregulates the JAK/STAT2 signaling pathway in gastric cancer AGS cells and inhibits JAK/STAT3 signaling pathway in gastric cancer AGS cells exhibits cytotoxic effects in cells in the MGC803, induces apoptosis, has antioxidant effects, reduces MMP and lower Blc-2 expression, increases caspase-3 expression	this effect is stronger when d-limonene with berberine is used simultaneously
Clove [44,45,113,120,121]	eugenol flavonoids: 5-hydroxy-7,4′-dimethoxy-6,8-di-C-methylflavone (eucalyptin), kaempferol 3-O-β-d-glucopyranoside, and kaempferol 3-O-α-l-rhamnopyranoside	shows an inhibitory effect on AGS gastric cancer cell proliferation inhibited the proliferation of human GC cells	induces apoptosis of cancer cells early; mainly causing a decrease in the S-phase population; induces caspase-8 and caspase-3 in the absence of p53; has anti-metastatic activities on AGS cell line independent of p53, P21, and SMAD4; inhibits the secretion of TGF-β type 2 isoform and intracellular expression of TGF-β. stops the G2/M phase of the cell cycle of human GC cells	Eugenol derivatives of β-aminoalcohol were more cytotoxic to A549 and AGS cells compared to β-alkoxyalcohol derivatives and the parent substance
Thyme [53,54,55]	thymol carvacrol chlorogenic acid	exhibits genotoxic and cytotoxic effects on AGS cells inhibited cell proliferation-induced DNA damage, apoptosis, and ROS production shows affinity for GC target genes, strong anticancer activity against various cancer and non-cancer cell lines	induces apoptosis by producing ROS and regulates the cell cycle by prolonging the sub-G1 cellular phase in AGS cells, damages MMPs and activates proapoptotic proteins; Bax; PARP; and caspase-7, -8, and -9, increases in caspase-3 exhibits antiproliferative effects and induction of apoptosis, which are regulated by Bax, Bcl-2, caspase-3, and caspase-9 proteins	there are different data on the effect on Bcl-2 expression; depending on the study, thymol has no effect on Bcl-2 expression or causes a decrease in it
Basil [122]	anthocyanin and flavonoid derivatives	cell death and inhibition of cell viability, cytotoxicity, antioxidant activity, apoptosis, reduced tumor growth, and cell cycle arrest	no exact data discovered	-

## Abbreviations

5-FU	5-fluorouracil
6-MITC	6-(methylsulfinyl)hexyl isothiocyanate
ACC	Acetyl CoA synthase
AGS	Gastric adenocarcinoma
AITC	Allyl isothiocyanate
AKT	Protein kinase B
Akt	Serine/threonine-protein kinase
ALP	Alkaline phosphatise;
AMPK	AMP-activated protein kinase
AP-1	Activator protein 1
ARF	Alternative reading frame
ARF	Reading frame
ATF1	Activating transcription factor 1
[B(a)P]	benzo(a)pyrene
Bax	Bcl2-associated X protein
Bcl-2	B-cell lymphoma 2
BUN	Serum urea nitrogen
CA	Cinnamaldehyde;
CagA	Cytotoxic-associated gene A
CasNPs	Casein nanoparticles
CASP1	Caspase-1
CASP3	Caspase 3
CASP8	Caspase 8
CASP9	Caspase 9
CDH1	Cadherin 1
CYR61	Cysteine-rich angiogenic inducer 61
EBV	Epstein–Barr virus
EBVaGC	EBV-associated gastric cancer
EO	Essential oil
ERK 1/2	Extracellular signal-regulated kinase 1/2
FASN	Fatty acid synthase
Foxm1	Forkhead box protein M1
GC	Gastric cancer
GCSCs	Gastric cancer stem cells
Gli1	GLI Family Zinc Finger 1
GSDMD	Gasdermin D
GSDME	Gasdermin E
GSH	Glutathione
GST	Glutathione-S-transferases
GSTP	Glutathione S-transferase P
*HATs*	*Histone* acetyltransferases
HDACs	Histone deacetylases
HER2	Human epidermal growth factor receptor 2
HIF-1α	Hypoxia-inducible factor 1-alpha
HMGCR	3-hydroxy-3-methylglutaryl-coenzyme A reductase
IFN-γ	Interferon gamma
IL	Interleukin
IL-1β	Interleukin-1β
IL-6	Interleukin-6
IL-8	Interleukin-8
IP	Intraperitoneal
JNK	C-Jun N-terminal kinases
KIF2C	Kinesin superfamily member 2C
KLF5	Krueppel-like factor 5
LC3 B	Light chain 3 B
LDH	Lactate dehydrogenase
MAPK	Mitogen-activated protein kinase
MESP1	Mesoderm posterior 1
mir	MicroRNA
MMP	Mitochondrial membrane potential
MMP-2	Matrix metalloproteinase-2
MMP-9	Matrix metalloproteinase-9
MNNG	Methyl-N-Nitro-N-Nitrosoguanidine
MyD88	Myeloid differentiation primary response 88
MYL9	Myosin regulatory light polypeptide 9
NCC	N-carboxymethyl chitosan
NCC-SLN	Curcumin-loaded modified solid lipid nanoparticles
NF-κB	Nuclear factor kappa B
NLRP3	NLR family pyrin domain containing 3
Nrf2	Nuclear factor erythroid 2-related factor 2
NSAIDs	Non-steroidal anti-inflammatory drugs
nsLTPs	Nonspecific lipid transfer proteins
OxR	Oxaliplatin-resistant
P-gp	P-glycoprotein
p-JAK2/JAK2	Janus associated kinase
p38 MAPK	p38 mitogen-activated protein kinases
PARP	Poly adenosine diphosphate-ribose polymerase
PCNA	Proliferating cell nuclear antigen
PDL-1	Programmed death-ligand 1
PI3K	Phosphatidylinositol-3 kinase
PI3K/Akt/mTOR	phosphatidylinositol-4,5-bisphosphate 3 kinase/protein kinase B/mechanistic target of rapamycin
PIK3R3	Phosphoinositide-3-kinase regulatory subunit 3
PIP	Piperine
PKM2	Pyruvate kinase muscle isozyme M2
PTT	partial thromboplastin time
ROS	Reactive oxygen species
RSK/CREB	ribosomal S6 kinase/cAMP response element binding protein
RSK2	ribosomal S6 kinase 2
SAE	Saffron aqueous extract
SFN	Sulforaphane
Shh	Sonic hedgehog homolog
shRNA	Specific short hairpin RNA
SMYD3	SET and MYN-domain containing 3
SN-38	7-ethyl 10-hydroxy camptothecin
SPREPB1	Sterol regulatory element-binding protein
STAT2	Signal transducer and activator of transcription 2
STAT3	Signal transducer and activator of transcription 3
TBX15	T-box transcription factor 15
TGF-β	Transforming growth factor beta
TIMP1	Tissue inhibitor of metalloproteinases
TLRs	Toll-like receptors
TNF	Tumor necrosis factor
TNF-α	Tumor necrosis factor α
TP53	Tumor suppressor protein p53 gene
TPM4	Tropomyosin 4
Uch-L1	Ubiquitin carboxy-terminal hydrolase isozyme L1
uPA	Urokinase plasminogen activator
uPAR	uPA receptor
VacA	Vacuolating cytotoxin A
VEGF	Vascular Endothelial Growth Factor
